# Preclinical Evaluation of Dimethyl Itaconate Against Hepatocellular Carcinoma *via* Activation of the e/iNOS-Mediated NF-κB–Dependent Apoptotic Pathway

**DOI:** 10.3389/fphar.2021.823285

**Published:** 2022-01-14

**Authors:** Anurag Kumar Gautam, Pranesh Kumar, Ritu Raj, Dinesh Kumar, Bolay Bhattacharya, P.S. Rajinikanth, Kumarappan Chidambaram, Tarun Mahata, Biswanath Maity, Sudipta Saha

**Affiliations:** ^1^ Department of Pharmaceutical Sciences, Babasaheb Bhimrao Ambedkar University, Lucknow, India; ^2^ Department of Pharmacology, Aryakul College of Pharmacy and Research, Lucknow, India; ^3^ Centre of Biomedical Research, SGPGIMS Campus, Lucknow, India; ^4^ Geethanjali College of Pharmacy, Hyderabad, India; ^5^ Department of Pharmacology and Toxicology, School of Pharmacy, King Khalid University, Abha, Saudi Arabia

**Keywords:** dimethyl itaconate, hepatocellular carcinoma, NF-κB, mitochondrial apoptosis, ^1^H-NMR–based metabolomics

## Abstract

Hepatocellular carcinoma (HCC) is one of the most common tumors affecting a large population worldwide, with the fifth and seventh greatest mortality rates among men and women, respectively, and the third prime cause of mortality among cancer victims. Dimethyl itaconate (DI) has been reported to be efficacious in colorectal cancer by decreasing IL-1β release from intestinal epithelial cells. In this study, diethylnitrosamine (DEN)-induced HCC in male albino Wistar rats was treated with DI as an anticancer drug. The function and molecular mechanism of DI against HCC *in vivo* were assessed using histopathology, enzyme-linked immunosorbent assay (ELISA), and Western blot studies. Metabolomics using ^1^H-NMR was used to investigate metabolic profiles. As per molecular insights, DI has the ability to trigger mitochondrial apoptosis through iNOS- and eNOS-induced activation of the NF-κB/Bcl-2 family of proteins, CytC, caspase-3, and caspase-9 signaling cascade. Serum metabolomics investigations using ^1^H-NMR revealed that aberrant metabolites in DEN-induced HCC rats were restored to normal following DI therapy. Furthermore, our data revealed that the DI worked as an anti-HCC agent. The anticancer activity of DI was shown to be equivalent to that of the commercial chemotherapeutic drug 5-fluorouracil.

## Introduction

Hepatocellular carcinoma (HCC) is one of the most common tumors affecting a large population worldwide, with the fifth and seventh greatest mortality rates among men and women, respectively, and the third leading cause of mortality among cancer victims (Hashimoto et al., 2009). Since the molecular pathway of HCC is still unknown to the scientific community, there are very few synthetic drugs available on the market to cure it (Keshari et al., 2017). This is recorded as most challenging scenario for the researchers. On the other hand, there are very few drugs available to manage HCC condition, and pharma companies have had little success due to chemotherapeutic resistance (Keshari et al., 2017). Sorafenib, as the only medication of high preference for HCC therapy, has a poor efficacy and is resistant to various molecular enzymes, making it a poor therapeutic option for the disease (Avila et al., 2006; Giannelli et al., 2014). Moreover, it also has a considerable number of distressing negative effects and is highly overpriced (Kondo et al., 2017). As a result, in order to improve HCC treatment options, novel compounds must be discovered and studied.

Recent literature suggested that a number of inflammatory mediators have crucial role in inflammation associated with HCC, and nuclear factor-κβ (NF-κβ) is one of the regulators for HCC progression (Guo et al., 2009; Alqahtani et al., 2019). In addition, it is postulated that nitric oxide is the important modulator for the activation of NF-κβ after phosphorylation in the presence of AKT enzyme (Bonavida and Baritaki, 2011). This activation is promoted in the presence of various nitric oxide synthetase enzymes (NOS), that is, inducible NOS (iNOS)/endothelial NOS (eNOS) at HCC sites (Bonavida and Garban, 2015). Finally, activated NF-κβ triggers the action of Bcl-2 family proteins, that is, pro-apoptotic (BAX and BAD) and antiapoptotic (Bcl-2 and Bcl-xl) in the presence of caspases, which ultimately leads to mitochondrial apoptosis (Marquardt and Edlich, 2019).

Dimethyl itaconate (DI) has been reported to be active against colorectal cancer *via* reducing the secretion of IL-1β from intestinal epithelial cells (Wang et al., 2020). A recent patent suggested that DI suppresses the iNOS gene expression at carcinogenic sites (Van Nest and Tuck, 2007), and it had protective effect against lipopolysacchride-induced mastitis *via* inhibiting the NF-κβ signaling pathway (Zhao et al., 2019). In this study, we wanted to explore the anti-HCC potential of DI *via* the e/iNOS-mediated NF-κB–dependent pathway.

Recent literature revealed that oxidative stress plays a vital role in HCC progression and growth, and diethylnitrosamine (DEN) produces cancer in liver tissue, that is, HCC (Kumar et al., 2015; Singh et al., 2018). Here, the anti-HCC potential of DI was measured by various molecular techniques such as the enzyme-linked immunosorbent assay (ELISA) and Western blot analysis to figure out the molecular mechanism behind the anti-HCC effect of DI. Later, we performed various histopathological and biochemical estimations to strengthen the hypothesis. Lastly, nuclear magnetic resonance (NMR)–based metabolomics were carried out to measure perturbed metabolites during HCC illness before and after therapeutic management with DI and to establish the metabolic pathway determination during HCC.

## Materials and Methods

### Drugs and Reagents

Genetix Biotech Asia Pvt. Ltd., New Delhi, India, and Thermo Fischer Scientific Ltd., Bangalore, India, supplied molecular biological assays and chemical reagents for the Western blot analysis. Additional chemicals and solvents were given by Himedia Ltd., Mumbai, India. Double distilled water (Borosil, India) was used throughout the study.

### Design of Experiments

Male albino Wistar rats weighing 110–130 g, aged 6- to 8-week old, were used for the experiment, with five groups, and each group containing a total of eight animals (n = 8) (Approval No 1648/PO/Re/S/12/CPCSEA/46). Animals were acclimatized to laboratory conditions (temperature [25 ± 1°C] with a 12 h dark/light cycle and free access to food and tap water) for 1 week prior to the start of the experiment. The groups of animals were distributed into five categories: Group 1 (Normal Control, NC) received 0.25% CMC (2 ml/kg), Group 2 (Carcinogen Control, CC) received N-nitrosodiethylamine (DEN, 100 mg/kg, i.p. once a week for total 6 weeks) (Kumar et al., 2019), Group 3 (Positive Control, PC) received DEN + 5-flurouracil (5-FU, 10 mg/kg, i.p. for total 15 days after HCC induction), Group 4 (Treatment 1) received DEN + DI (T1, 100 mg/kg, orally for total 15 days after HCC induction), and Group 5 (Treatment 2) received DEN + DI (T2, 200 mg/kg, orally for 15 days after HCC induction) (Singh et al., 2018). The DEN dosages were determined using previously reported studies (Kumar P et al., 2020) and DI ECHA (2021).

### Estimation of Physiological Parameters

Changes in the body weight were recorded on the first and last day of the study, and the % weight increase was calculated. The weight of liver and % of tumor incidence were determined to evaluate the cytotoxic effects in untreated and treated experimental groups.

The enzyme levels of alanine aminotransferase (ALT), aspartate aminotransferase (AST), lactate dehydrogenase (LDH), and alkaline phosphatase (ALP) were measured in serum by using commercially available kits (Yadav et al., 2015; Chauhan et al., 2017; Rai et al., 2018). Catabolic by-products, including biliverdin and bilirubin, were identified in the liver tissue (Miranda et al., 2001). All experimental procedures are thoroughly explained in the Supplementary Material.

Various serum lipid profiles, including triglycerides (TG), total cholesterol (TC), and high-density lipoproteins (HDL), were calculated using a commercial lipid profile kit (Agappe Diagnostic Ltd., Kerala, India). Friedewald’s method was used to calculate low-density lipoprotein (LDL) and very–low-density lipoprotein (VLDL) (Maurya et al., 2019). All experimental methods are thoroughly explained in the Supplementary Material.

LDL(mg/dL)=TC−HDL−(TG/5),

VLDL(mg/dL)=TC−HDL−LDL.

The histological methodology utilized has previously been described (Yadav et al., 2015; Chauhan et al., 2017; Rai et al., 2018; Rai, 2019) and is also included in the Supplementary Material.

### Determination of Total Nitrite/Nitrate Content and NOS Activity Assay

Miranda et al. (2001) used a method modified by Motawi et al. (2008) to quantify the total nitrite/nitrate content in stomach tissue homogenates (Basak et al., 2020).

### Estimation of Cytokines by ELISA

Rat ELISA kits from Invitrogen Bioservices India Pvt. Ltd. (Thermo Fisher Scientific), Bengaluru, India, were used to determine eNOS, iNOS, NF-κβ, and cytochrome-C levels (CytC). Caspase-3 and caspase-9 were supplied by Geno Technology Inc., Noida, India. ELISA was used to assess hepatic tissues in accordance with the manufacturer’s instructions (available online) (Kumar et al., 2019; Maurya et al., 2019).

### Western Blot Analysis

Immunoblotting has been used to determine the amounts of Bcl-xl, Bcl-2, BAD, and BAX protein expression. All antibodies were given by Thermo Fisher Scientific, Waltham, MA, United States. After lysing cells in a radio-immunoprecipitation assay (RIPA) buffer and spinning at 10,000 rpm speed for 15 min at 4°C, the protein levels were tested using the Bradford reagent. Proteins (50 mg) were electrophoresed on a polyacrylamide gel containing 12% sodium dodecyl sulfate (SDS) and then transferred to a PVDF membrane. The PVDF membrane was blocked in 5% skimmed milk containing phosphate-buffered saline (PBS) with 0.1 percent Tween 20 (PBS-T) for 3 h at 4°C before being probed up overnight at 4°C along with primary antibodies (1:500 dilution of each) obtained from Cell Signaling Technology, MA, United States: Bcl-2 (D17C4), Bcl-xL (54H6), and BAX (D3R2M). TBS-T was used to rinse the PVDF membranes thrice. The next day, they were treated for 3 h at room temperature with anti-rabbit secondary antibodies conjugated to horseradish peroxidase at 1:3,000 dilutions. After washing the film thrice with TBS-T, the PVDF membrane was developed using enhanced chemiluminescence ECL (PierceTM ECL Western Blotting Substrate), and the images were recorded using Chemidoc (Chakraborti et al., 2018; Basak et al., 2020; Chakraborti et al., 2020).

### ^1^H-NMR–Based Metabolomics Spectroscopy

The samples were prepared using the previously described methodology (Rai et al., 2018; Kumar U et al., 2020). The NMR spectra were collected using a Bruker BioSpin Avance-III 800 MHz NMR spectrometer set to Cryoprobeat 298K. According to the previously reported protocol, the blood sample was placed in the NMR-sealed tube (5 mm) with TSP of known concentration. The standard Bruker’s pulse program library sequence (CPMGPR1D) was employed to obtain transverse relaxation–edited CPMG (Carr–Purcell–Meiboom–Gill) NMR spectra for every blood sample, which comprised of a pre-saturated water peak irradiated during a 5 s recycling delay (RD).

The residual peaks were allocated using data from the Biological Magnetic Resonance Data Bank database (BMRBD) and the Human Metabolome Database (HMD), which included previously reported NMR spectra of substances (Wishart et al., 2007; Ulrich et al., 2007). The stored data were analyzed for chemometric characteristics using the web-based online server named MetaboAnalyst (version 3.0; publicly accessible web server for academic use: https://www.metaboanalyst.ca/) (Xia et al., 2009; Puig-Castellví et al., 2016). MetaboAnalyst was used to import the modified metabolite data, which were then analyzed using the over representation approach (ORA). The *Rattus norvegicus* metabolic library was subjected to route topology analysis and pathway enrichment using the hypergeometric test and degree centrality approaches. Based on the pathway topology and pathway enrichment analyses, the pathway analysis module computed an impact factor and a fit coefficient (p) for each analyzed route. The findings were presented with *p*-values (from route enrichment analysis) in the *y*-axis, the intensity of color and impact values (from pathway topology analysis) in the *x*-axis, and the size of the circle, with the most influential pathway in blue (Quan-Jun et al., 2015). The metabolomics methodology is described in full in the Supplementary Material.

### Statistical Data Analysis

GraphPad Prism software 5.0 (San Diego, CA, United States) was used to analyze statistical data. The data were presented as a mean standard deviation (SD) (*n* = 8). The statistical data were evaluated using one-way ANOVA (analysis of variance), followed by Bonferroni’s multiple comparison test. There were statistically significant differences between the carcinogen control and test groups (*p0.05, **p0.01, ***p0.001).

## Results

### Estimation of Various Physiological Parameters

**FIGURE 1:**
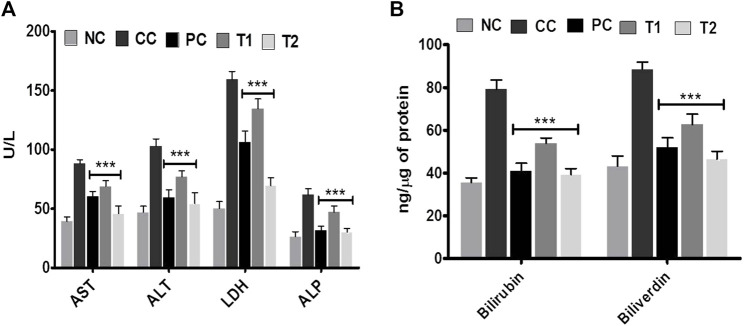
Effects of DI after oral administration of 100 mg/kg and 200 mg/kg for 15 days in carcinogen control rats. **(A)** Enzyme levels of AST, ALT, LDH, and ALP in serum and **(B)** catabolic by-product (bilirubin and biliverdin). Data are represented as mean ± SD (*n* = 8). Statistically significant differences were observed between carcinogen control and test groups [one-way ANOVA followed by the Bonferroni multiple comparison test (***p < 0.001, **p < 0.01, *p < 0.05)]. The studied groups are (NC, Normal Control; CC, Carcinogen Control; PC, Positive Control, T1, DI (100 mg/kg) and T2, DI (200 mg/kg).

At two distinct dosages of DI, that is, 100 (T1) and 200 (T2) mg/kg, we simultaneously assessed numerous liver parameters such as the body and liver weight as well as the tumor incidence number. The body weight and tumor incidence numbers were revealed to be significantly different in DEN-treated rats, but they turn normal following DI treatment (Table 1). As physiological parameters, liver enzymes such as ALT, AST, LDH, and ALP were examined and recorded. All enzyme levels in CC rats were considerably higher before treatment as well as significantly lower after treatment, especially at T2 dosages (Figure 1A). This is the primary indication of protective action of DI.

**TABLE 1:** DI chemotherapy effects on liver markers in rats with DEN-induced carcinogenesis (NC, Normal Control; CC, Carcinogen Control; PC, Positive Control; T1: DI (100 mg/kg) and T2: DI (200 mg/kg).

S. No	Groups	Initial day body weight (g)	Final day body weight (g)	Weight variation (g)	Liver weight (g)	Tumor incidence no
1	NC	110.00 ± 3.15	125.00 ± 3.03	15.00 ± 0.88	4.15 ± 0.16	0.00 ± 0.00
2	CC	131.00 ± 5.01	103.00 ± 5.00	-28.00 ± 1.52	9.56 ± 0.25	51.00 ± 2.54
3	PC	122.00 ± 2.99	128.00 ± 3.01***	6.00 ± 1.11***	4.58 ± 0.97***	10.00 ± 1.02***
4	T1	109.00 ± 3.65	113.00 ± 3.05***	4.00 ± 0.88***	6.03 ± 0.96**	21.00 ± 0.80***
5	T2	116.00 ± 3.12	128.00 ± 3.55***	12.00 ± 1.32***	4.11 ± 0.68***	8.00 ± 1.01***

The data are provided as mean ± SD (n = 8). Between the CC and test groups statistically significant differences were found [one-way ANOVA, followed by the Bonferroni multiple comparison test] (*p < 0.05), **p < 0.01 and ***p < 0.001)].

In addition, the effects of DI on catabolic pigments, including bilirubin and biliverdin, on liver tissues were investigated (Figure 1B). DI normalized the level of these two pigments (*p* < 0.001) that were elevated 2.2-folds in CC groups. This response was more significant at T2 doses and was equivalent to conventional chemotherapeutics, 5-FU. The lipid profile had been an innovative metric for measuring the protective impact of DI. In continuation with observed results in Table 2, we found that there was an increase in TC, TG, LDL, VLDL levels and a decrease in the HDL level in CC rats. Both TC and TG levels were improved to normal, and LDL and VLDL levels were normalized after treatment. In contrast, the HDL level in serum was improved ∼two-folds as compared to normal.

**TABLE 2:** Rat serum lipid profiles were used to assess the effects of DEN and DI treatment (NC, Normal Control; CC, Carcinogen Control; PC, Positive Control; T1: DI (100 mg/kg) and T2: DI (200 mg/kg).

S. No	Parameters	NC	CC	PC	T1	T2
1	TC (mg/dl)	159.68 ± 9.68	236.2 ± 7.95	173.51 ± 5.83***	189.15 ± 6.12***	165.26 ± 5.69***
2	TG (mg/dl)	95.76 ± 5.23	184.57 ± 7.53	124.85 ± 6.58***	146.84 ± 6.98***	112.79 ± 6.12***
3	HDL (mg/dl)	68.25 ± 7.65	27.59 ± 7.98	50.87 ± 7.31***	42.63 ± 5.41**	58.52 ± 6.59***
4	LDL (mg/dl)	19.15 ± 2.62	36.91 ± 1.98	24.97 ± 2.79***	29.36 ± 1.75***	22.55 ± 1.29***
5	VLDL (mg/dl)	72.27 ± 5.27	171.69 ± 2.12	97.67 ± 6.32***	117.15 ± 5.12***	84.18 ± 5.13***

The data are provided as mean ± SD (n = 8). Between the CC and test groups statistically significant differences were found [one-way ANOVA, followed by the Bonferroni multiple comparison test] (*p < 0.05), **p < 0.01 and ***p < 0.001)].

### Histopathology of Liver Tissues

**FIGURE 2:**
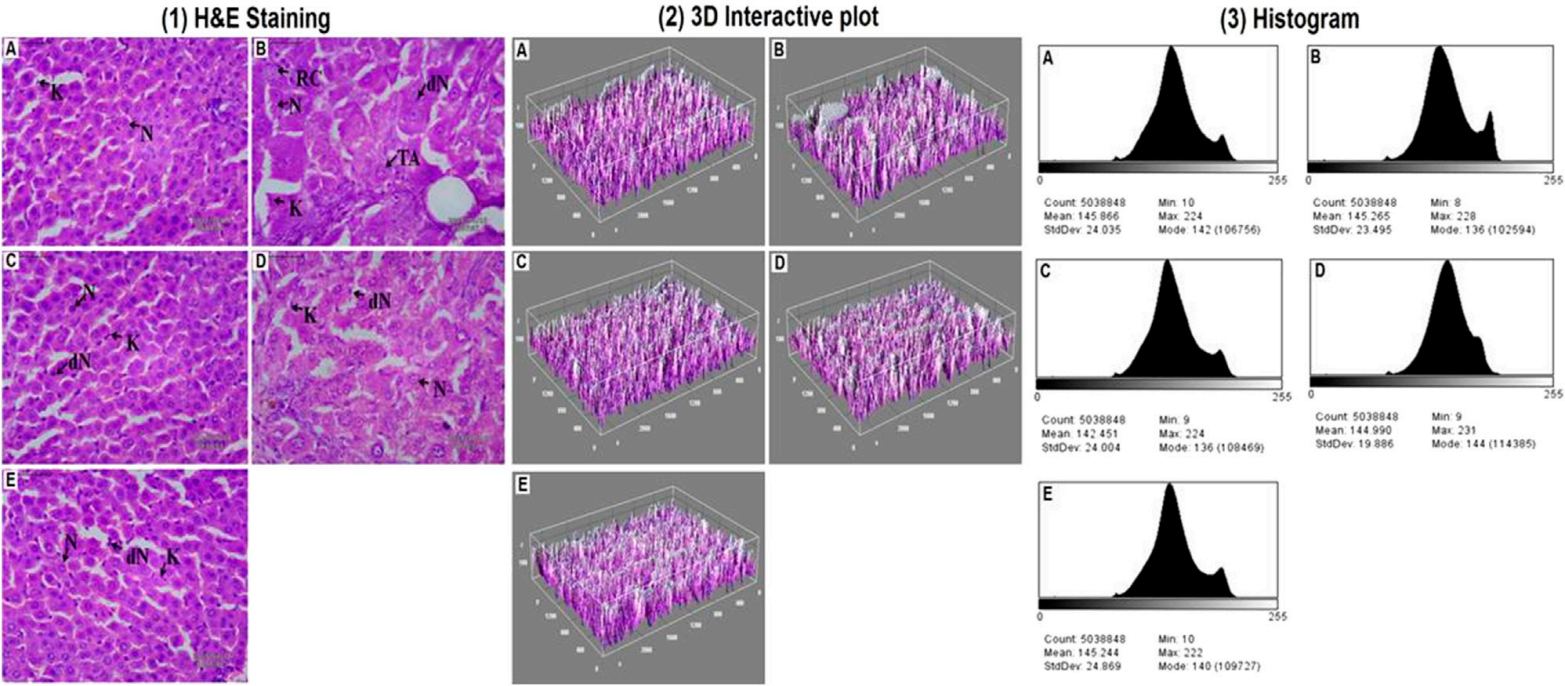
Hepatic pathological changes in DEN-induced rats. [1] Histopathological changes (40X, Scale bar 50 μm) and the studied groups are A: Normal Control, B: Carcinogen Control, C: Positive Control, D: DI (100 mg/kg) and E: DI (200 mg/kg). The abbreviations used are normal nucleus (N), degenerated nucleus (dN), ruptured hepatic cells (RC), and tumor anaplastic cells (TA). 3D image reconstruction and software-based analysis dataset of constructs representing the score was done by using ImageJ (NIH) software by thresh holding of stained zones of H&E images followed by pixel vs intensity determination by the [2] 3D interactive surface plot and [3] the histogram analysis.

Histopathological alterations in the liver tissue were also examined using hematoxylin and eosin (H&E) stains before and after chemotherapy. An examination of the tissues revealed that in the DEN-treated group, normal nucleus (N), degraded nucleus (dN), ruptured hepatic cells (RC), and tumor anaplastic cells (TA) were seen (Figure 2). After DI therapy, the damages in the liver tissue were repaired. Later, by thresh holding stained zones of H&E images, followed by pixel vs intensity determination by the 3D interactive surface plot and log-histogram analysis, a 3D image reconstruction and software-based analysis dataset of constructs representing score was further documented *via* ImageJ (NIH) software. ImageJ analysis further emphasized the results obtained from H&E stains (Figure 2).

### Mechanistic Exploration of Antineoplastic Properties of DI

We used colorimetric tests of nitrite/nitrate concentrations and NOS activity in hepatic tissue to investigate the DI influence on cancer-related NO-based inflammatory events.

**FIGURE 3:**
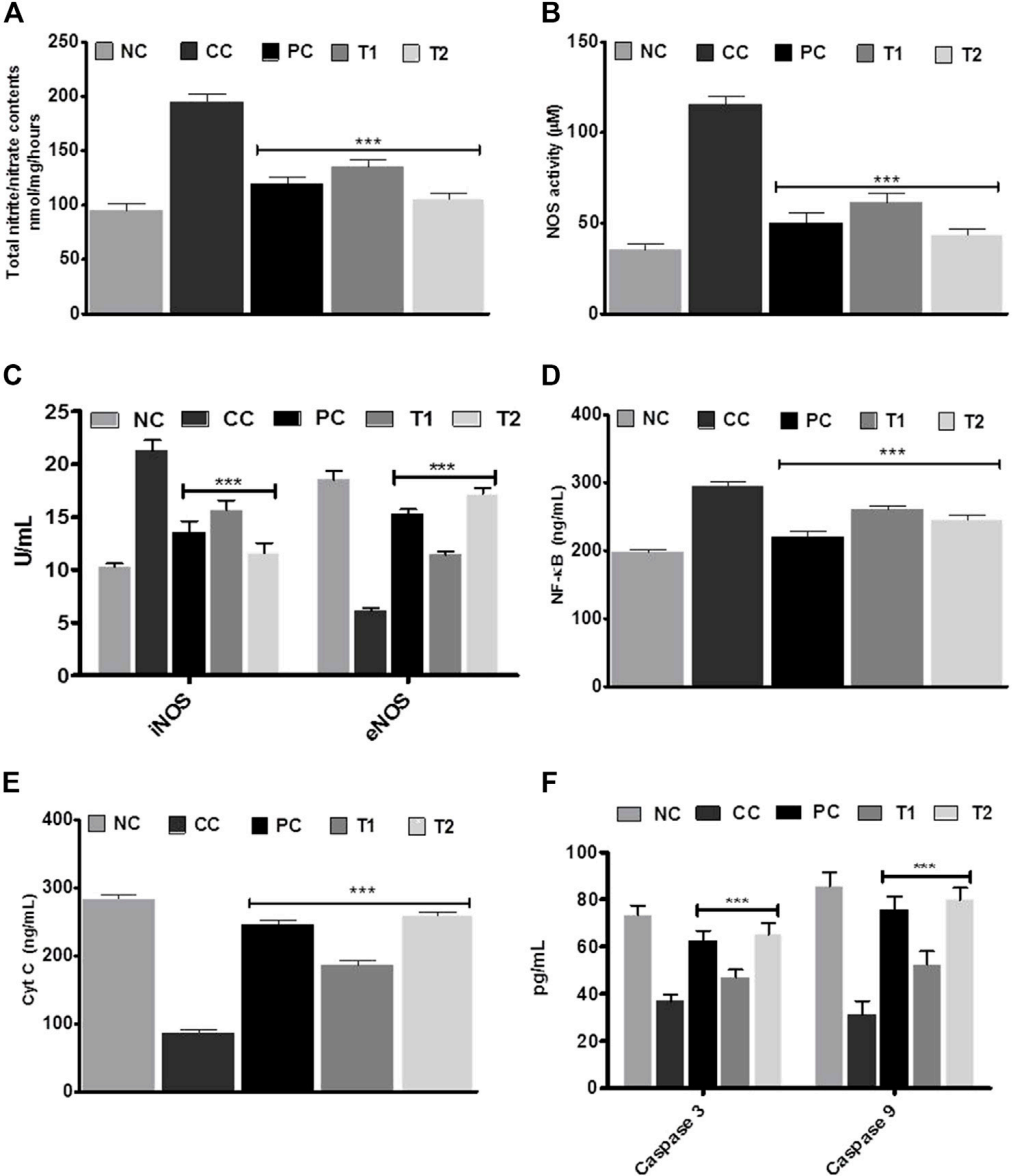
**(A)** Total nitrite/nitrate contents **(B)** nitric oxide synthase (NOS) activity assay. **(C)** iNOS and eNOS, **(D)** NF-xB **(E)** Cyt C, and **(F)** caspase-3 and caspase-9 enzymes activities in liver tissue before and after treatment with DI. Data are represented as mean + SD (n-8). Statistically significant differences were observed between carcinogen control and test groups [one-way ANOVA followed by Bonferroni multiple comparison test (***p < 0.001, **p < 0.01, *p < 0.05)] The studied groups are NC, Normal Control; CC, Carcinogen Control; PC, Positive Control; T1, DI (100 mg/kg) and T2, DI (200 mg kg).

The DEN-treated group promoted a strong initiation of nitrite/nitrate contents and NOS activity, indicating HCC condition (Figures 3A,B). After taking DI orally, either of individuals returned to normalcy. Later, the ELISA experiment was performed to test the versatile conditions of iNOS, eNOS, NF-κB, caspase-3, and caspase-9. The CC group exhibited an induction in iNOS with a corresponding reduction in eNOS in liver tissue. After oral administration of DI, both iNOS and eNOS concentrations were effectively normalized in the treatment group (Figures 3C). NF-κB showed the same pattern as iNOS (Figure 3D). However, when CC rats were compared to NC group rats, the concentration of apoptotic markers such as cyt C and caspases (3 and 9) were decreased accordingly ∼3.0 and ∼2.0 fold. DI therapy restored normality markedly, with a more apparent impact at 200 mg/kg, which was equivalent to the amount of 5-FU (Figure 3E, F).

**FIGURE 4:**
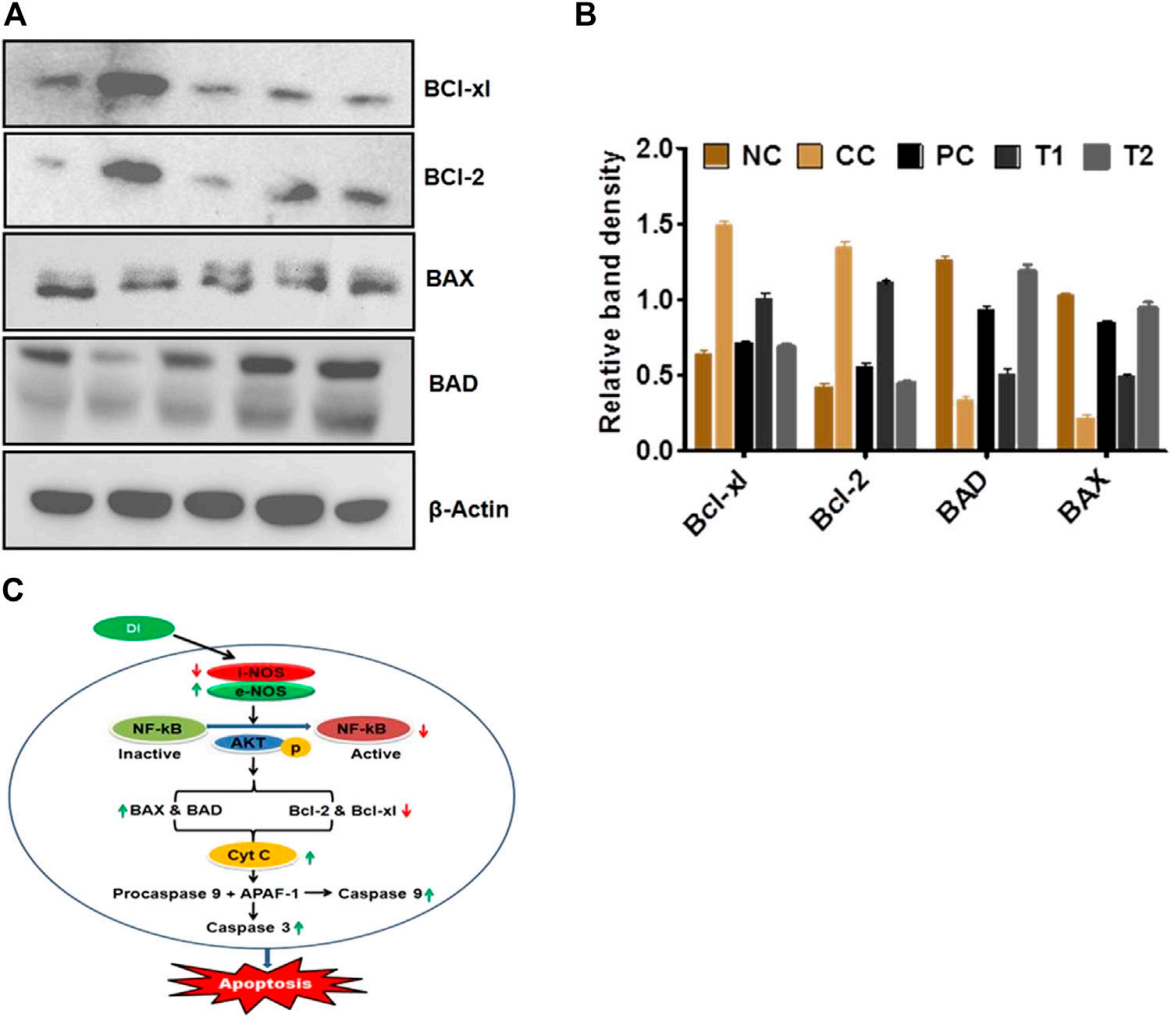
**(A,B)** Protein expression levels of BAD, BAX, Bel-xl, Bcl-2, and β-actin in liver tissue after treatment with DI (determined by quantitative Western blot analysis). **(C)** Plausible mechanism of anticancer activity of DL. Data are represented as mean SD (*n* = 8). Statistically significant differences were observed between carcinogen control and test groups (one-way ANOVA followed by the Bonferroni multiple comparison test (***p < 0.001, **p < 0.01, *p < 0.05)). The studied groups are NC, Normal Control; CC, Carcinogen Control; PC, Positive Control; TI, DI (100 mg/kg) and T2, DI (200 mg/kg).

Western blot studies were used to determine the concentrations of protein expression of BAX, BAD, Bcl-xl, and Bcl-2. In DEN-treated rats, the of BAX and BAD proteins content were reduced, whereas Bcl-xl and Bcl-2 proteins content were increased. Furthermore, reverse trend expressions were further documented after 5-FU and DI treatments dose (Figures 4A–C).

Throughout this investigation, β-actin worked likely as a surveillance protein (Supplementary Figures S1–S5).

### Biochemical Alterations of Metabolites Through ^1^H-NMR–Based Metabolomics Studies

Metabolic alterations during HCC condition were further investigated using 1D ^1^H-CPMG NMR spectra and also to demonstrate the protective efficacy of DI before and after treatments.

**FIGURE 5:**
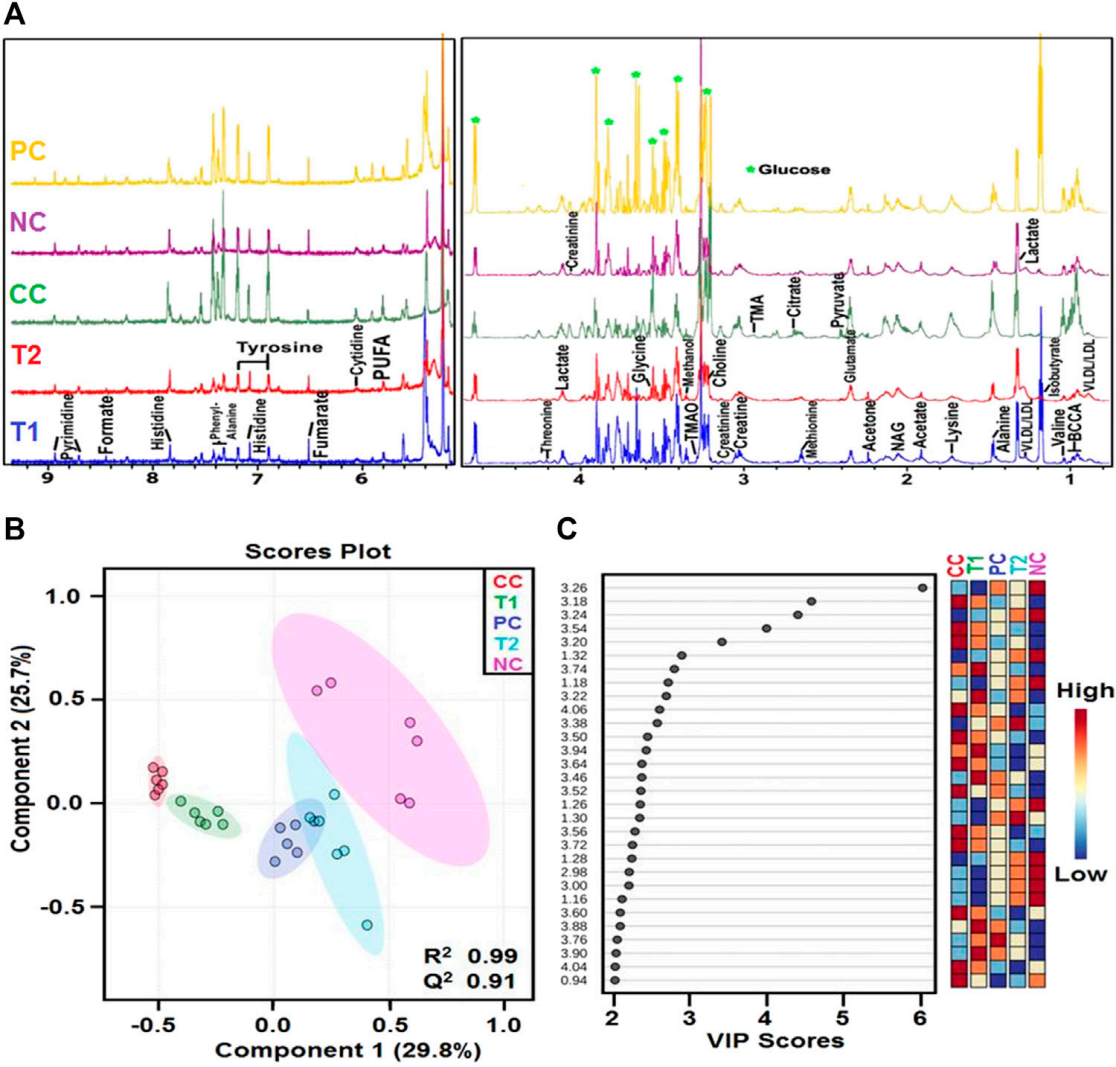
**(A)** Stack plot of representative ID H CPMG NMR spectra of rat serum obtained from different groups NC, Normal Control; CC, Carcinogen Control; PC, Positive Control; TI, DI (100 mg kg) and 12, DI (200 mg kg) and **(B)** OPLS-DA analysis: The 2D OPLS-DA analysis of ID H CPMG NMR spectra score plot derived from combined analysis comprising of all the groups. NC, Normal Control; CC, Carcinogen Control; PC, Positive Control; T1, DI (100 mg kg) and T2: DI (200 mg/kg). **(C)** The potential discriminatory metabolite entities identified from VIP scores derived from OPIS-DA modeling of complete data matrix and resulted VIP scores for top 30 metabolite entities are shown in increasing order of VIP score values to highlight their discriminatory potential.

**FIGURE 6:**
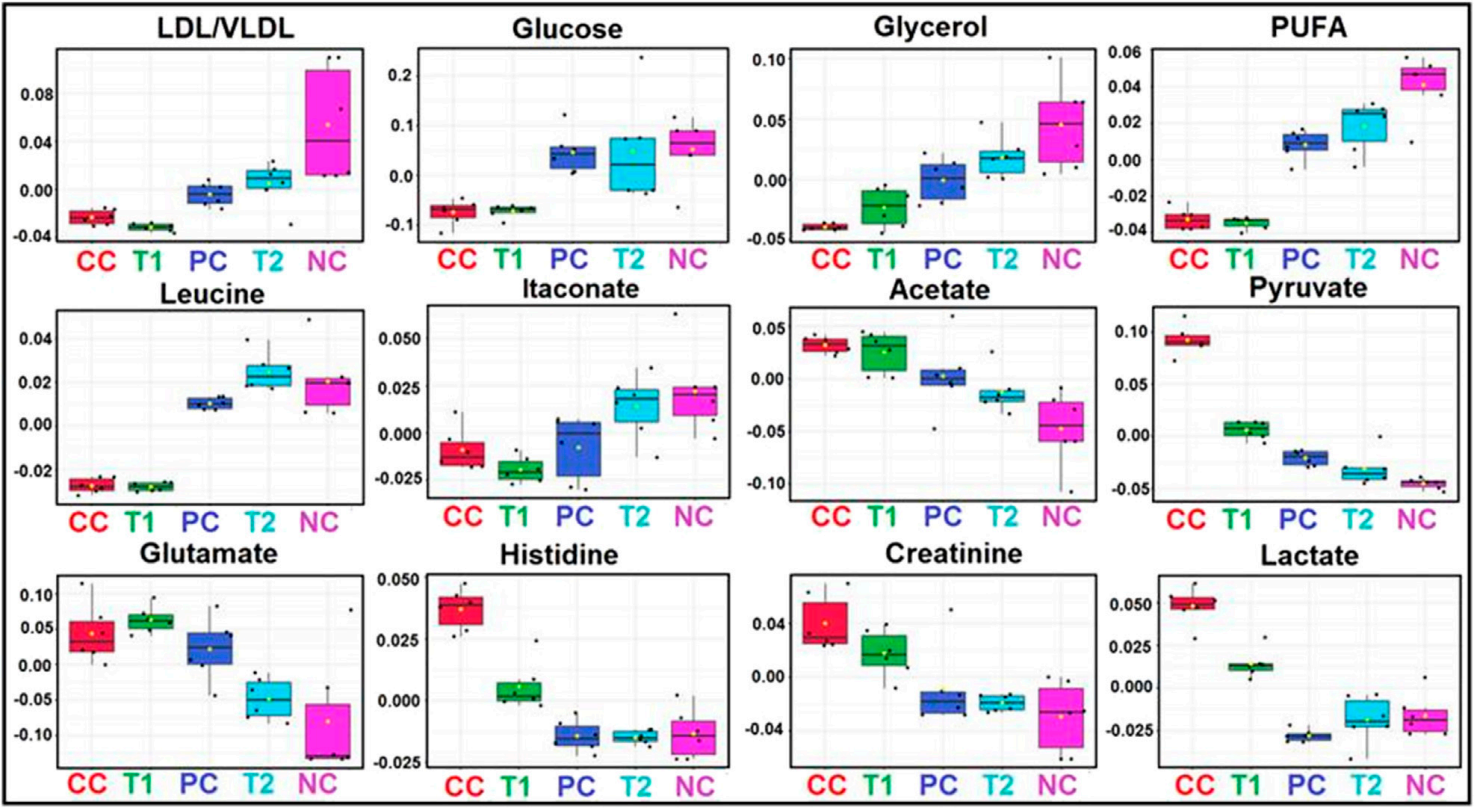
Metabolic effects of DI treatment: The box-cum-whisker plots are showing relative variations in quantitative profiles of serum metabolites relevant in the context of the pathophysiology of hepatic cancer. In the box plots, the boxes denote interquartile ranges, horizontal line inside the box denote the median, and bottom and top boundaries of boxes are 25th and 75th percentiles, respectively. Lower and upper whiskers are 5th and 95th percentiles, respectively. NC, Normal Control; CC, Carcinogen Control; PC, Positive Control; T1, DI (100 mg/kg) and T2, DI (200 mg/kg).

The 1D ^1^H-CPMG NMR spectra obtained from the rat serum sample of different control and treatment groups are shown in Figure 5A. The lipids/lipoproteins (e.g., low-density lipoprotein (LDL), very low-density lipoprotein (VLDL), and poly-unsaturated fatty acids (PUFAs)) and amino acids (e.g., leucine, glutamate, and histidine) were detected in the ^1^H-NMR spectra of rat serum samples. Lactate, pyruvate, acetate, creatine, glycerol, glucose and itaconate (Note: bin at 3.14 ppm is considered as itaconate signal here overlapping with other metabolic signals as no evident 1H NMR signal corresponding to its methylene proton seen near 5.5 ppm) were some of the prominent metabolites (Figure 6).

First, the ^1^H-NMR dataset was prepared to explore metabolic perturbations between control and carcinogenic rats, and later, statistical data modeling and analysis were further performed using multivariate analysis tools in MetaboAnalyst (Boyault et al., 2007; Khan et al., 2011). A principal component analysis (PCA) was used to analyze with the help of the analytical quality system and to investigate apparent outliers. Again, the orthogonal partial least squares data analysis (OPLS-DA) model would provide a summary of the entire descriptive collection of samples and help detect factors that are responsible for the differences between individuals. The nature of OPLS-DA model was optimized with two factors, that is, Q^2^ and *R* ^2^. Significant metabolic alteration between normal and DEN-treated carcinogenic groups was presented through a combined and pair wise analysis of OPLS-DA data which revealed *R* ^2^ = 0.99 and Q^2^ = 0.91 (Figure 5B). There was a significant decrease in LDL/VLDL glucose, glycerol, PUFA, leucine, and itaconate and an increase in acetate, pyruvate, glutamate, histidine, creatinine, and lactate (Figure 6). After 5-FU and DI therapy, we have seen a normalization of all these metabolites. NMR-based metabolomics again established practical separation of metabolites among the groups on the *X*-axis which was also confirmed by Score plots acquired from 1D ^1^H-CPMG NMR spectra (Figure 5B) (from OPLS-DA and S plots). The VIP score plots in Figure 5C show the separation of distinct groups, and the combined correlations of 2D OPLS-DA and VIP score analyses revealed substantial variations in metabolic profiles across diverse groups. When the statistically substantial threshold of variable effect on projection (VIP) values derived from the OPLS-DA model was larger than 1.0, the top 12 metabolite sensitizers were carefully picked. Meanwhile, the regulated peak area’s *p*-values from two-tailed Student’s t tests were declared statistically significant (^*^p < 0.05). The log2 fold change (FC) method was used to show how these very diverse metabolites differed across all of the groups investigated. When the *p*-value is less than 0.05, the findings are deemed statistically significant. In Figures 5, 6, some suitable metabolic changes are shown as univariate box plots.

**FIGURE 7:**
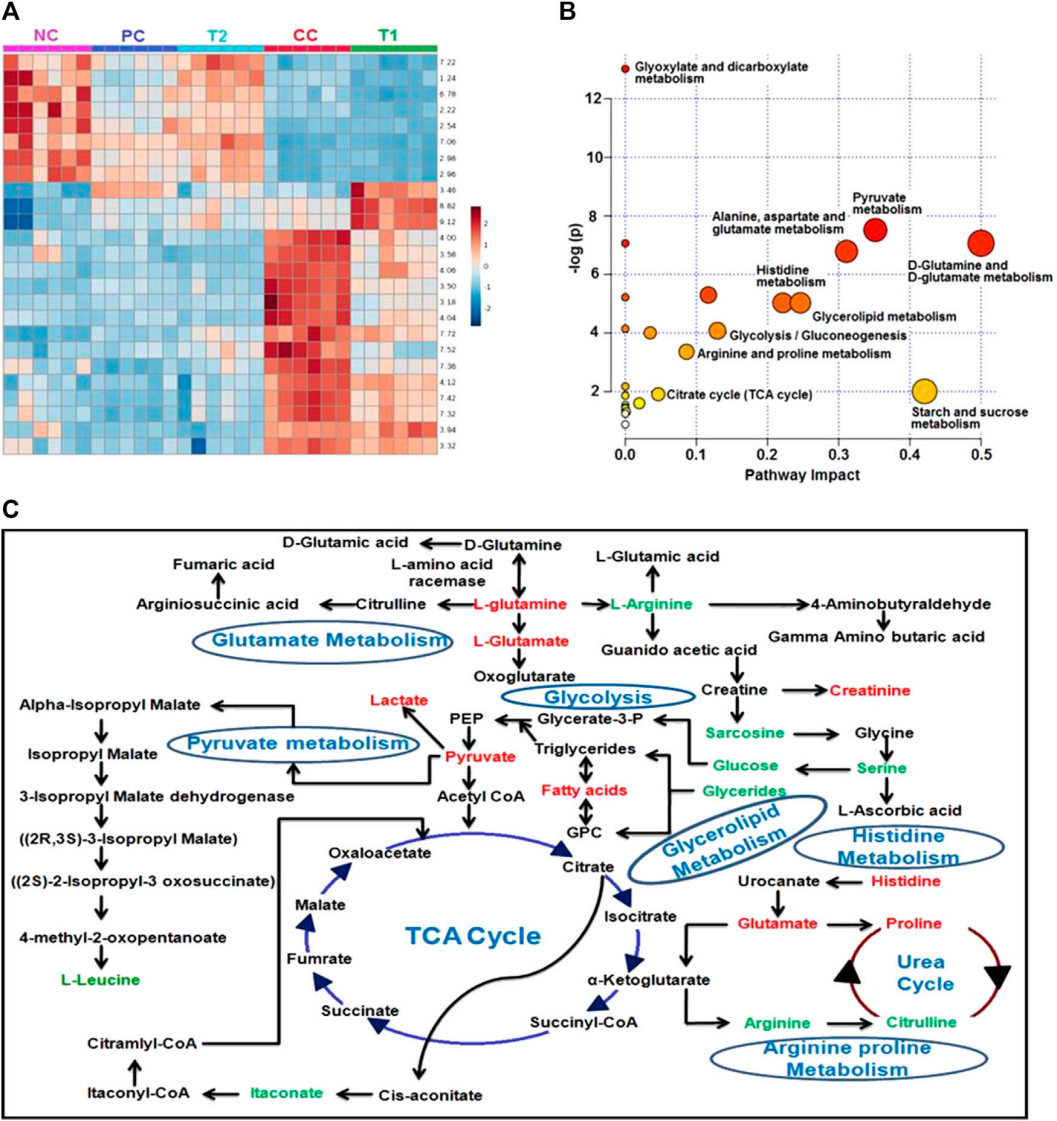
**(A)** Heatmap of significant metabolites represented their concentration changing pattern. **(B)** Shows the module that supports pathway analysis of integrating enrichment analysis and pathway topology analysis and visualization for one model organisms. Pathways impact is shown the highly significant pathways of major metabolites. **(C)** Major metabolic pathways of the most relevant metabolites after DI treatments and CC group involved in glycolysis, glutamate metabolism, pyruvate metabolism, glycerolipid metabolism, Krebs cycle, the urea cycle, and histidine metabolism. Red: increased concentration profile of particular metabolites in the CC group. Green: decreased concentration profile of particular metabolites in the CC group.

The DI-treated group had metabolomic profiles that were very comparable to the control group, according to heat mapping of each dataset (Figure 7A). A number of specific metabolites relevant to HCC exhibit substantial changes in response to the CC group rats, which were partially reversed by 5-FU and DI therapies. These single metabolites may subsequently be incorporated into a variety of important metabolic pathways (Figures 7B,C). Glycolysis, the Krebs cycle, ketogenesis, β-oxidation, lipolysis, and the urea cycle metabolism were all altered by DI therapy in our dataset.

## Discussion

Numerous studies have emphasized on the critical function of NO in tumor etiology and development, including the involvement of multiple signaling pathways throughout cancer malignancy. Several NO responses have been reported during tumor growth (Singh and Gupta 2011). Over expression of iNOS has led to angiogenesis and metastasis of cancerous cells. On the other hand, eNOS has an opposite trend and over expression of eNOS reduces angiogenesis and metastasis which ultimately lead to cellular apoptosis (Xu et al., 2002). Later, NF-κβ may be over expressed due to activation of eNOS/iNOS at the HCC site, and phosphorylation of NF-κβ may be taken place in the presence of AKT enzymes which results in the conversion of inactivated NF-κβ to its active form (Bonavida and Garban, 2015). In continuation, activated NF-κβ triggers the mitochondrial apoptotic pathways, that is, activation of Bcl-2 family proteins [pro-apoptotic (BAX and BAD) and antiapoptotic (Bcl-2 and Bcl-xl)] in the presence of caspases (Xu et al., 2002; Singh and Gupta 2011).

DEN-induced hepatocarcinogenesis is an ideal model for HCC as the formation of HCC is similar to cancer formation in the human liver (Boyault et al., 2007; Khan et al., 2011). In our study, HCC formation in rats using DEN was supported by the increased concentration of ALT, AST, and ALP in serum, and hepatocarcinogenesis was histopathologically identified (Iddamaldeniya et al., 2003; Iddamaldeniya et al., 2006). After the formation of HCC, DI was administered in two doses, and the antineoplastic activity was checked and compared with the positive control, that is, 5-FU. During molecular observation, the ELISA assay revealed that the total nitrate/nitrite content and NOS activity were decreased in T2 doses of DI which indicated the primary defense mechanism of DI. In the next ELISA experiment, we found that DI improved eNOS and reduced iNOS concentrations. As described in the previous paragraph (Singh and Gupta 2011), these actions convey the message that DI plays a crucial role in the NO-based cellular apoptosis mechanism. In HCC condition, both nitrate/nitrite and NOS were released due to the physiological mechanism of the body; indeed, eNOS/iNOS action triggered and finally balanced after DI treatment.

It has been postulated that NOS imbalances trigger the release of NF-κβ at the molecular level, followed by phosphorylation in the presence of AKT enzyme (Seki et al., 2007; Guo et al., 2009; Bonavida and Baritaki, 2011; Khan et al., 2011). Furthermore, it is widely known that cellular apoptosis happens through two mechanisms, namely, the extrinsic death receptor system and the intrinsic mitochondrial pathway. The Bcl-2 family regulates the mitochondrial pathway, which releases both pro-apoptotic (BAX and BAD) and antiapoptotic (Bcl-2 and Bcl-xl) proteins. All of these apoptogenic signals are thought to cause the release of CytC from mitochondria, which then connect with procaspase 9 and APAF1 to create an apoptosome (Rang et al., 2011; Sanaei and Kavoosi, 2021). In the presence of caspase-3 and caspase-9, this apoptosome causes apoptosis. As a result, the Western blot analysis demonstrated that DI restored normality by upregulating Bcl-2 and Bcl-xl and downregulating BAX and BAD. DI restored the lowered levels of caspase-3 and caspase-9 in the latter stages, suggesting that it might be an essential apoptosis activator. When taken simultaneously, DI promotes apoptosis by activating the NF-κβ–mediated Bcl-2 family proteins → CytC→ Caspase-3 and Caspase-9 signaling cascade *via* iNOS and eNOS.

This anticancer property of DI was further confirmed by various physiological, biochemical, and histopathological assays. The carcinogenic condition of rats was established through the decrease in body/liver weight and higher formation of carcinogenic nodules. The normalization of these conditions after treatments with 5-FU and DI were the key signatures of the preventive potential of DI. Furthermore, tumor-induced hyperlipidemia condition promotes hepatic cirrhosis during HCC that eventually results in liver damage and failure (Festi et al., 2004; Huang et al., 2016). Damage of the liver during hyperlipidemia in HCC rat sera was further established by higher concentrations of TC, TG, LDL, VLDL and lower concentration of HDL. This action was again accentuated using DI oral administration, protective action documented in the similar experiment. In contrast, various liver enzymes, including ALT, AST, and ALP, are indicators of liver damage during pathophysiological condition of the liver (Green and Flamm 2002; Kweon et al., 2003). As a result, when the liver is damaged, all of these enzymes seep into the bloodstream, causing their levels in the bloodstream to rise. The protective potential of DI was measured by the observed effectiveness of DI in restoring these enzymes to normal levels. Similarly, LDH is the non-specific indicator of DEN-induced HCC (Green and Flamm 2002; Kweon et al., 2003). The similar effect was found as in ALT and AST, which ultimately indicated the protective ability of DI. Furthermore, an increased level of catabolic liver pigments such as bilirubin and biliverdin further demonstrated liver damage during HCC (Green and Flamm 2002). The elevation of these pigments and normalized after oral treatment also specify the antineoplastic property of DI against HCC. CC rats also had vacuole development and abnormally shaped nuclei and cytoplasm, according to histological examinations (Kumar et al., 2019; Maurya et al., 2019). This vacuole formation was decreased after DI and 5-FU treatments, and HCC condition was improved and clearly observed through histopathology.

The ^1^H-NMR–based metabolomics analyses provide a concrete idea about metabolic signature quantitatively during HCC condition before and after treatments of DI. Identification of the aberrant metabolites in HCC condition may help in early detection and treatment of the disease (Frederich et al., 2016). This helps create a potential HCC pharmacological action map in the context of metabolomic pathways (Griffin and Shockcor, 2004). We used ^1^H-NMR–based serum metabolomics to show, for the first time to our knowledge, the impact of DI on HCC. The metabolic alterations generated by DEN and how DI assisted were investigated using NMR-based metabolite profiling and multivariate statistical data analysis. Amino acids, ketone bodies, choline metabolism, glycolysis, the TCA cycle, phosphatidylinositol, and gluconeogenesis are all part of the HCC metabolic pathway (Vander Heiden, 2011; Cuperlovic-Culf et al., 2016).

We observed the Warburg effect in NMR-based metabolic studies where an increased level of lactate and decreased level of glucose in CC rat sera with respect to NC rats. This observation was similar to previously published data in the glycolytic pathway where lower concentration of glucose was found due to higher consumption of glucose by the cancerous cells and formation of lactate as by-product (Asgari et al., 2015). In addition, PUFA and VLDL/LDL are the precursor of cholesterol formation which is the important constituent of cell membrane production (Liu et al., 2014). A decreased level of PUFA and VLDL/LDL in CC rats again demonstrated carcinogenic condition in the liver. DI orally administration again restored this metabolite to normalcy, and thus presenting significant data of DI’s anticancer activities.

The box-cum-whisker plots revealed that amino acids like glutamate and leucine were consumed by HCC cells to form the cell membrane and mature the cellular constituents (Gao et al., 2009). Because these metabolites were absorbed at a quicker rate by HCC tissues, we discovered lower levels of them. Both metabolites, on the other hand, were observed to be raised following DI and 5-FU treatments. Glycerol concentrations in box-cum-whisker plots were reduced once more, indicating an increase in the lipid metabolism during HCC (Vander Heiden, 2011; Rahman and Hasan, 2015). Treatment with 5-FU and DI also indicated a greater quantity of glycerol in rat serum, indicating that these drugs have the capacity to improve glycerol metabolism. In addition, DI therapy stabilized blood levels of itaconate, pyruvate, acetate, creatinine, and histidine, and DI’s protective effect was demonstrated in a comparable experiment.

## Conclusion

The molecular mechanism of HCC is still unclear to researchers, hence finding a new anti-HCC medicine is very challenging. In light of the present HCC therapeutic situation, we suggested DI as an anti-HCC drug for the first time, along with its molecular mode of action. The significance of DI in HCC therapy might be explained by the stimulation of caspase-mediated mitochondrial apoptosis, according to the findings of this research. This activation is triggered by the NF-κβ pathway, which is mediated by eNOS/iNOS. DI has the ability to cause mitochondrial apoptosis through iNOS- and eNOS-induced activation of the NF-B/Bcl-2 family of proteins → CytC→ Caspase-3 and Caspase-9 signaling cascade, according to both ELISA and Western blot investigations. Later on, NMR-based serum metabolomics confirmed the metabolic changes before and after DI therapy, which helped explain DI’s cellular activity once again. As a result, we may infer that DI is a superior anti-HCC agent for future drug design considerations.

## Data Availability

The datasets presented in this study can be found in online repositories. The names of the repository/repositories and accession number(s) can be found in the article/Supplementary Material.
