# Supplementary material for: Preclinical Evaluation of Dimethyl Itaconate Against Hepatocellular Carcinoma via Activation of the e/iNOS-Mediated NF-κB–Dependent Apoptotic Pathway
Source: Front Pharmacol. 2022 Jan 14;12:823285. doi: 10.3389/fphar.2021.823285 (PMC8795766; doi:10.3389/fphar.2021.823285)

**Supplementary Data Sheet**

**Preclinical evaluation of dimethyl itaconate against hepatocellular carcinoma via activation of e/iNOS mediated NF-κB dependent apoptotic pathway**

Anurag Kumar Gautam^1*^, Pranesh Kumar^1,2^, Ritu Raj^3^, Dinesh Kumar^3^, Bolay Bhattacharya^4^, P.S. Rajinikanth^1^, Kumarappan Chidambaram^5^, Tarun Mahato^3^, Biswanath Maity^3^, Sudipta Saha^1^

^1^Department of Pharmaceutical Sciences, Babasaheb Bhimrao Ambedkar University, VidyaVihar, Raibareli Road, Lucknow 226025, India

^2^Department of Pharmacology, Aryakul College of Pharmacy & Research, Gauri- Bijnaur Road, Natkur, Adjacent to CRPF Base Camp, Lucknow, Uttar Pradesh, 226002, India

^3^Centre of Biomedical Research, SGPGIMS Campus, Raebareli Road, Lucknow 226014, Uttar Pradesh, India

^4^Gethanjali college of Pharmacy, Cheeryal, Keesara, Hyderabad, 501301, India

^5^Department of Pharmacology & Toxicology, School of Pharmacy, King Khalid University, Abha 62529, Saudi Arabia

**^*^Correspondence:**

Anurag Kumar Gautam

Email: [kumargautamanurag@gmail.com](mailto:kumargautamanurag@gmail.com)

**Estimation of Serum ALT and AST**

Serum ALT and AST were estimated according to previously described method, with slight modifications and performed by using ALT and AST assay kit obtained from Excel Diagonistic Pvt. Ltd., Hyderabad, India. Working solutions were prepared by dissolving 4 ml of reagent 1 (mixture of L-alanine and α-ketoglutarate for ALT and mixture of L-aspartate and α-ketoglutarate for AST) and 1 ml of reagent 2 (mixture of nicotinamide adenine di neucleotide phosphate, NADP and lactate dehydrogenase, LDH) and kept at 2 to 8°C for future use. The whole assay was performed in 96-well plate and each plate contained 0.5 ml of working solution and 0.05 ml of serum. This was incubated for 1 minute at 37°C and change in optical density (ΔA340/min) was measured per minute for the next 3 min using the UV/VIS Spectrophotometer (Labtronics, Australia). Data was calculated by the following equations:

ΔA_340_/min = [A_340_ (Time 2) - A_340_ (Time 1)] / [Time 2 (min) - Time 1 (min)]

ALT or AST Activity (unit/ml) = ΔA_340_/min × 1746 × 10^-3^

**Serum LDH**

Serum LDH estimated according to previously described method, with slight modifications and performed by using LDH assay kit obtained from Agappe Diagonostic Pvt. Ltd., Kerala, India. Working solutions of R_1_ of 4 mL (Tris buffer, pyruvate and sodium chloride) and R_2_ of 1 mL were mixed together and kept at 2 to 8°C for further use. 10 µL of serum was mixed with 1000 µL of working solution and this was incubated for 1 minute at 37°C and change in optical density (ΔA340/min) was measured per minute for the next 3 min using the UV/VIS Spectrophotometer (Labtronics, Australia). Data was calculated by the following equations:

LDH (U/L) = (ΔOD/min)X 16030

**Serum ALP**

ALP was estimated according to method described previously, with slight modifications and performed by using ALP assay kit obtained from Excel Diagonistic Pvt. Ltd., Hyderabad, India. All groups contained 1 ml buffer substrate and 3 ml distilled water. Later, 0.1 ml distilled water, 0.1 ml phenol and 0.1 ml serum was added to blank, standard and test groups, respectively. All groups including control groups are incubated for 15 min at 37°C. 0.1 ml serum was added to control group after incubation. All the tubes were mixed properly and absorbance was measured at 510 nm wavelength of light.

ALP activity (unit/ml) = [A (Test) – A (Control) / A (Standard) – A (Blank)] × 7.1 × 10^-3^

**Tissue bilirubin**

Bilirubin in liver was measured as per the following procedure published earlier in the literature with slight modifications. All the tissue samples were thawed and homogenized in phosphate buffer saline (8.0 g sodium chloride, 0.2 g potassium chloride, 0.2 g potassium dihydrogen phosphate_,_ 1.15 g disodium hydrogen phosphate_,_ 0.372 g ethylenediaminetetraacetic acid disodium salt, pH 7.4). 500 µL of tissue homogenate (10%) was added to 2.0 mL of 1.5% butylatedhydroxy toluene in acetone:ethanol (1:1) in a eppendorf tube. Simultaneously, fresh diazo reagent was prepared by mixing 300 µL of 10% sodium nitrite and 8.0 mL of 2M p-toluene sulfonic acid, then combining 4.0 mL of this mixture with 2.0 mL of 2.1% p-iodoaniline in glacial acetic acid, kept at room temperature for 2.0 min. Then this solution was diluted with distilled water (10 mL) and 200 µL of 1.5M ammonium sulfamate. This working diazo reagent was kept on ice for 5min and 500 µL was added to each sample homogenates. Diazo blank reagent was freshly prepared by combining 2 mL of p-toluene sulfonic acid and 5.0 mL of 10% ascorbic acid, followed by addition of 2.1% p-iodoaniline in glacial acetic acid and 2.0 mL of n-butyl acetate, mixed and used immediately. Finally, all the tubes were incubated for 1 hour on ice in dark. After incubation freshly prepared 3.0 mL of 1% ascorbic acid in 0.1 M sodium chloride was added to each vial. All the vials were shaken vigorously, kept for 1.0 min and centrifuged at 2400 rpm for 10 min. The absorbance of the upper organic phase was taken at 530 nM wavelength. The content of bilirubin was calculated as follows:

A_530_sample – A_530_ sample blank = ΔA_530_Test

**Tissue biliverdin**

Estimation of biliverdin was performed as per the method prescribed in the previous literature with slight modifications. Tissue samples were homogenized in phosphate buffer saline as per described in the previous section. 500 µL of tissue homogenate (10%) was combined with 500 µL of 10 M glacial acetic acid, 400 µL of 40 mM ascorbic acid, 500 µL of double distilled water and 100 µL of 200 mM barbituric acid. Samples were incubated in a water bath at 95^ᵒ^C in dark and then samples were extracted with butanol, vortexed and centrifuged. The upper organic layer was carefully removed and extracted with 2.5 mL 2M sodium hydroxide. The absorbance of upper layer was taken at 535 nM wavelength. The content of biliverdin was calculated as follows:

A_535_sample – A_535_ sample blank = ΔA_535_Test

**Lipid Profile assays**

Serum lipid profiles, including total cholesterol and triglycerides, were determined using a lipid profile kit (Agappe Diagnostic Ltd., Kerala, India), according to the manufacturer’s instructions. Briefly, 10 µL of the serum or standard was added to 1 mL of the working solution. The mixture was incubated for 5 min at 37°C, and the absorbance was measured at 505 nm. Cholesterol/triglyceride concentrations were calculated according to the following equation:

Cholesterol/triglyceride concentrations (mg/dL) = (Absorbance of serum/Absorbance of standard) × 200 For high density lipoprotein (HDL) measurement, HDL unesterified cholesterol was prepared by adding sulfated α-cyclodextrin and lipoproteins. Then, cholestenone and H_2_O_2_ were added to the reaction mixture. 5-Aminophenazone was then added to the reaction mixture, and the absorbance was measured at 600 nm. H_2_O_2_ formation was directly proportional to the concentration of HDL in the serum. Low density lipoprotein (LDL) and very low density lipoprotein (VLDL) concentrations were estimated by using Friedewald's formula, as follows:

LDL (mg/dL) = TC – HDL – (TG/5)

VLDL (mg/dL) = TC – HDL – LDL

**Histopathological studies**

Histopathological studies were also performed to find out the morphological changes of liver cells after PYZ and its metabolites administration. Liver tissues from each group were assessed for their morphological changes using haematoxyline and eosin staining. The tissues were preserved in 10% formalin overnight. Next day, the cells again were superseded by 70% isopropanol overnight. Later, the tissues were exposed to isopropanol at various concentrations (70, 90 and 100%) and dehydrated by 100% xylene. The tissue samples were then embedded in bees wax and 5μm sections were prepared by using microtome. Then, the tissues were succeeded by haematoxyline and eosin staining and observed under microscope.

**^1^H-NMR based metabolomics spectroscopy**

In the 1D ^1^H-CPMG NMR spectra, the peaks, chemical shifts were identified and assigned by comparing with the database library of Chenomx 8.1 software NMR suite (Chenomx Inc., Edmonton, Canada).

The multivariate data analysis was performed using the ^1^H-CPMG. All the procured ^1^H-NMR spectra were phasedmanually and the baseline corrected using TopSpin3.0 (Bruker NMR data Processing Software). For the analysis, the CPMGspectra were stored and automatically integrated utilizing the AMIX package (Version 3.8.7, Bruker, BioSpin).

The outliers were identified by using the principal component analysis. Partial least squares discriminate analysis with orthogonal signal correction (OPLS-DA) was used to demonstrate the disparities between the groups and also to identify the metabolites which play key role in group separation. R^2^ and Q^2^ in PLS-DA models play vital role in the validation of OPLS-DA models. Mann-Whitney test for pairwise comparisons were employed for estimation of statistical significance. Univariate analysis was employed for area under the curve measurement and box plot representation. This parameter helps in envisaging the dissimilarity in the levels ofconsiderably altered and discriminatory capability of metabolites as prospective biomarkers. A value close to 1 is often considered better in the classification. For determining the *p*-value along with up-regulation and down-regulation of metabolites, T-test univariate analysis was utilized.

The biochemical pathway was analyzed by utilizing the pathways library available in the metaboanalyst server for *Rattus norvegicus*. The altered metabolites in the obtained samples were identified through PLS-DA analysiswith a good VIP scores and compared with CC. The pathway analysismodule provided the information of affected metabolic pathways. The MetaboAnalyst software comprises of inbuilt functions of pathway topology analysis and enrichment analysis. The software do not recognizeslipid metabolites and membrane such as lipids,VLDL, LDL, HDL, OAG, NAG, etc., and are therefore were excluded from the analysis.

**Fig. S1** Full blot of β Actin

**
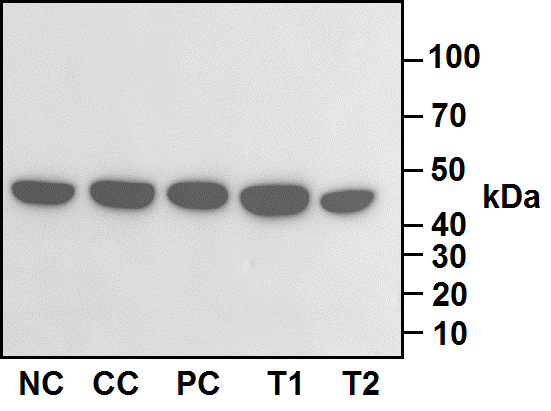
**

**Fig. S2** Full blot of BAD


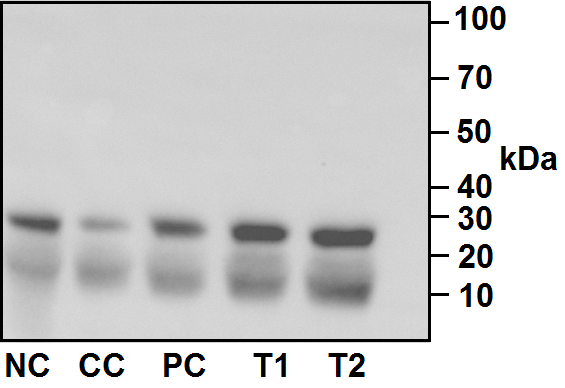


**Fig. S3** Full blot of BAX


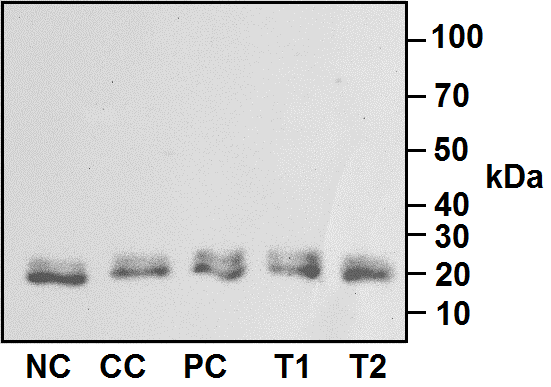


**Fig. S4** Full blot of Bcl-2


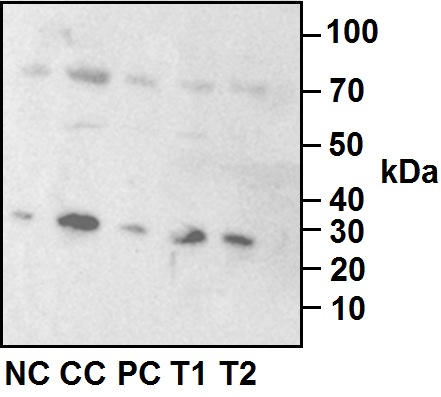


**Fig. S5** Full blot of Bcl-xl


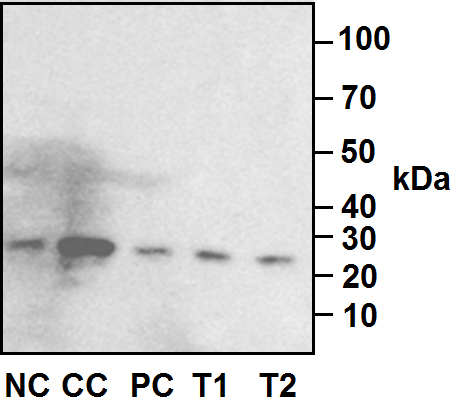

Supplement: Supplementary file 1 [file DataSheet1.docx]
